# Monogenic obesity due to MC4R deficiency: lessons from a multigenerational case

**DOI:** 10.1186/s40348-025-00214-z

**Published:** 2026-01-05

**Authors:** Eleni Z. Giannopoulou, Stefanie Zorn, Melanie Schirmer, Stephanie Brandt-Heunemann, Julia von Schnurbein, Claudia Nestoris, Abubakar Moawia, Reiner Siebert, Christian Denzer, Martin Wabitsch

**Affiliations:** 1 Department of Pediatrics and Adolescent Medicine, Division of Pediatric Endocrinology and Diabetes, University Medical Center Ulm, Ulm, Germany; 2 Department of Pediatrics and Adolescent Medicine, Division of Pediatric Endocrinology and Diabetes, Center for Rare Endocrine Diseases, Ulm University, Ulm, Germany; 3 German Center for Child and Adolescent Health (DZKJ), Partner Site Ulm, Ulm, Germany; 4 Endokrinologikum Hannover, Hannover, Germany; 5 Institute of Human Genetics, Ulm University and Ulm University Medical Center, Ulm, Germany

**Keywords:** Monogenic obesity, Liraglutide, Melanocortin 4 receptor

## Abstract

**Background:**

Melanocortin 4 receptor (MC4R) deficiency is the most common monogenic cause of obesity, yet remains underdiagnosed. Patients with monogenic obesity often undergo a frustrating diagnostic and therapeutic odyssey of years of ineffective lifestyle interventions before a causal diagnosis is made. We report a four-generation family where genetic testing in a child identified a likely pathogenic *MC4R* variant also carried by three ancestors.

**Methods:**

The studied family included a 7-year-old index patient, her mother, grandmother, and great-grandmother with a history of early-onset obesity. Panel sequencing of monogenic obesity genes was performed in the index patient whereas in the relatives targeted analysis of the familial *MC4R* variant was performed by Sanger sequencing.

**Results:**

The index patient developed severe obesity by age 2 years, with hyperphagia, tall stature, and dyslipidemia. Despite lifestyle interventions, her body mass index (BMI) continued to increase. At the age of 7 years, genetic panel testing identified a rare monoallelic variant in the *MC4R* gene c.913C > T; p.Arg305Trp, previously shown to impair receptor function. Treatment with liraglutide (3.0 mg/day) was initiated at age 8 years, resulting in marked reduction in BMI during the first year of treatment. Subsequent genetic testing of family members identified the same variant in her mother, grandmother, and great-grandmother, all of whom had a history of early-onset obesity and related comorbidities, consistent with segregation of the variant within the family.

**Conclusions:**

This case underscores the importance of early genetic testing in severe childhood obesity to avoid ineffective treatments and enable targeted therapies (e.g., GLP-1 analogues). Diagnosing (likely) pathogenic *MC4R* variants can also identify at-risk relatives, providing psychological and clinical benefits across generations.

## Background

Monogenic obesity refers to a group of rare, early-onset forms of obesity and accounts for up to 7% of patients with severe pediatric obesity [1]. Monogenic obesity is inherited in a Mendelian pattern, is typically early-onset and severe, and involves either chromosomal deletions or single gene defects [2]. Pathogenic variants in several genes have been found to be responsible for monogenic obesity, including the leptin gene (*LEP*), the leptin receptor gene (*LEPR*), the melanocortin-4 receptor gene (*MC4R*), proopiomelanocortin gene (*POMC*), the proprotein convertase subtilisin/kexin type 1 gene (*PCSK1*), the single-minded homolog 1 gene (*SIM1*), the SH2B adaptor protein 1 gene (*SH2B1)*, the melanocortin receptor accessory protein 2 gene* (MRAP2)* and others [2]. These genes are mainly involved in the hypothalamic leptin–melanocortin pathway, which plays a major role in the central regulation of hunger and satiety [2]. Because of this, patients with monogenic obesity typically exhibit extreme hyperphagia from early childhood, making it a key criterion — alongside early-onset severe obesity — for recommending genetic testing following current pediatric guidelines [3, 4].

Establishing a genetic diagnosis in obesity transforms clinical management by enabling pathway-specific therapies where available, and guiding selection of conventional pharmacologic agents based on underlying pathophysiology. Accordingly, targeted pharmacological therapies, such as metreleptin for patients carrying biallelic *LEP* variants and setmelanotide for patients with biallelic *LEPR*, *POMC*, and *PCSK1* variants, are available for selected monogenic obesity forms [5–7]. In addition, identifying the cause of severe obesity can lessen the feelings of guilt and blame for the patients and their families, and alleviate social stigma and discrimination.

Despite advances in genetic testing, monogenic obesity remains underdiagnosed due to limited testing availability and incomplete knowledge of its genetic forms, often resulting in prolonged diagnostic evaluations before confirmation [8]. The condition's low prevalence, phenotypic overlap with polygenic obesity, and variable access to genetic testing underscore the need for evidence-based selection criteria for genetic evaluation not only in pediatric, but also in adult obesity care [9, 10]. The aim of this case report is to describe a family spanning four generations affected by monogenic obesity, in which the underlying genetic cause was only identified after the youngest descendant underwent genetic testing, leading to a further diagnosis in three other affected family members.

## Patients and methods

**Fig. 1:**
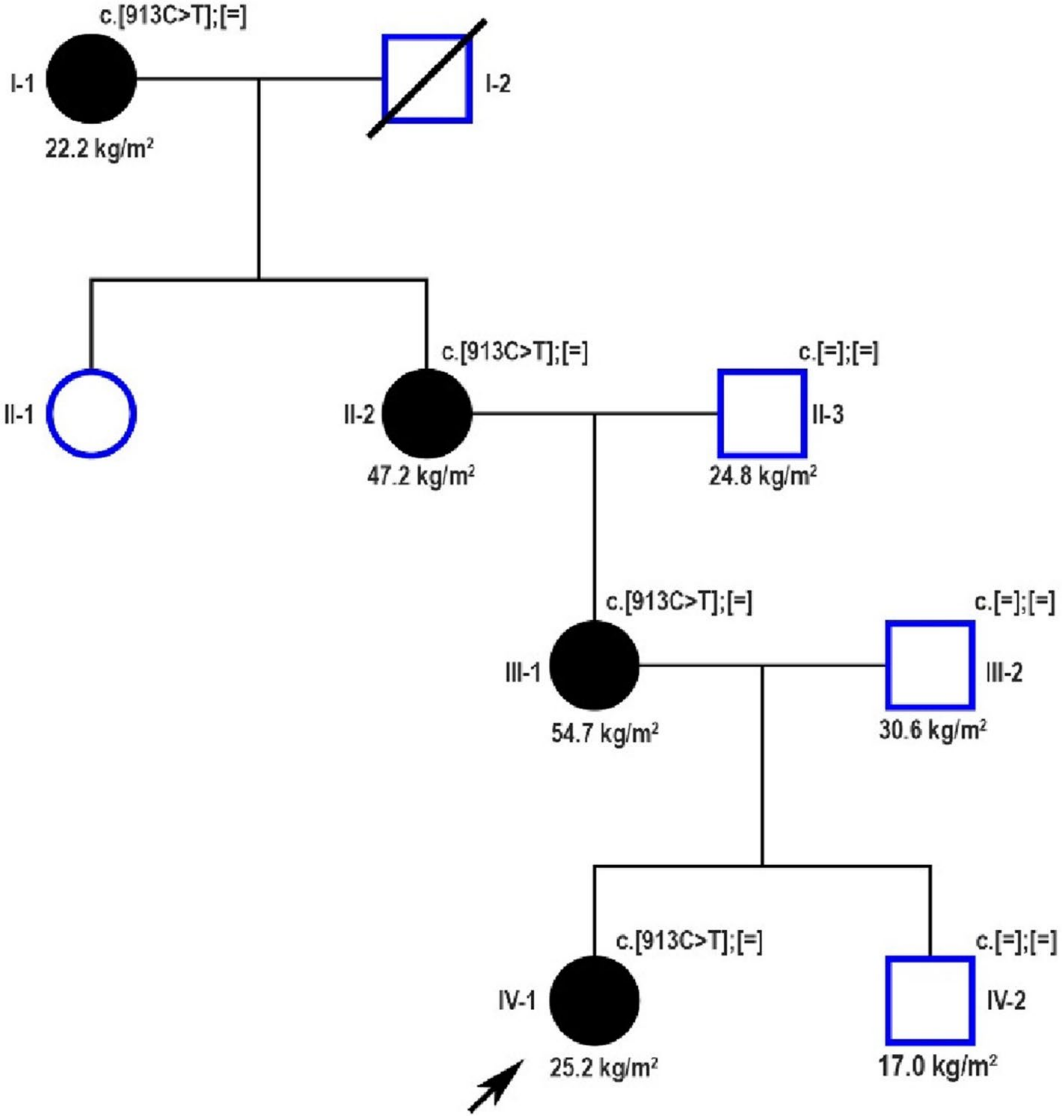
Pedigree of the reported family with severe early-onset obesity due to a likely pathogenic monoallelic MC4R variant. Squares represent males and circles females. A shape with a diagonal line means deceased. Filled black symbols indicate members with a history of severe early-onset obesity. The index patient is marked with a black arrow. The numbers beneath the symbols represent each family member's actual BMI value

We present a family of German origin in which severe, early-onset obesity has been reported over four consecutive generations in four female family members (Fig. 1). The index patient, her mother, grandmother and great-grandmother were examined at the Division of Pediatric Endocrinology and Diabetes, Department of Pediatrics and Adolescent Medicine, University Medical Center in Ulm, Germany and clinical data of the included patients were collected retrospectively.

Body mass index (BMI) was calculated by dividing weight (in kilograms) by the square of height (in meters). In order to design BMI trajectories from birth, previous anthropometric data (height and weight) were obtained from the index patient’s health booklet (German U Screenings provided by the German national health system) or previous medical records. Pediatric obesity was classified as a BMI > 97th percentile and severe obesity as a BMI > 99.5th percentile for age and sex according to the national guidelines for obesity in children and the German growth reference data [11, 12]. BMI standard deviation score (SDS) was calculated using the least mean squares method based on German growth references [11, 12]. Regarding adult patients, a BMI > 25 kg/m^2^ was defined as overweight and a BMI > 30 kg/m^2^ as obesity, according to the World Health Organization. Severe obesity in adults was defined as BMI > 35 kg/m^2^. Signs of hyperphagia were assessed by the Hyperphagia Questionnaire developed by Dykens et al. for the index patient [13] and based on self-reports for adult patients. The Dykens’ Hyperphagia Questionnaire is a 13-item parent/caregiver-completed tool assessing a child’s eating behavior and symptom severity. It covers three domains—behavior, drive, and severity—evaluating actions like food seeking or stealing, attitudes such as tantrums or food obsession, and the overall intensity of these behaviors. Each item is rated from 1 (no problem) to 5 (severe/frequent problem), except Item 12, which records age of onset. Hyperphagia is indicated by a total score above 19 [13]. Dyslipidemia was defined as the presence of one or more lipid levels (total cholesterol, LDL-cholesterol, HDL-cholesterol, non-HDL-cholesterol or triglycerides) [14–16].

For molecular genetic diagnosis, genomic DNA was isolated from blood or buccal swaps. In the index patient, panel sequencing of the genes *LEP*, *LEPR*, *MC4R*, *MC3R*, *PCSK1* and *POMC* was performed via Next-Generation sequencing (NGS) in a commercial lab (Hannover, Germany) whereas in the relatives targeted analysis of the familial *MC4R* variant was performed by Sanger sequencing using routine protocols (Institute of Human Genetics, Ulm, Germany). Genomic DNA was extracted from the buccal swab samples using DNA purification reagent kit (MACHEREY–NAGEL, Germany, Catalogue number: 740952.50). To confirm the missense variant c.913C > T; p.Arg305Trp in exon 1 of the *MC4R* in all available samples, PCR was performed with the forward primer 5′- CTGGGCCCCATTCTTCCTCCACT-3′ and reverse primer 5′-ACGGAAGAGAAAGCTGTTGCAGAAGTA-3′. PCR amplification was performed using AmpliTaq Gold DNA polymerase (Applied Biosystems). The PCR products were purified using Exo-SAP PCR Clean-up kit (Fermentas, Germany), sequenced bidirectionally using the BigDye Terminator v3.1 Cycle Sequencing Kit (Applied Biosystems, Foster City, CA, USA) and analyzed on the ABI Prism Applied Biosystems™ 3500xL Dx Genetic Analyzer (Thermo Fischer Scientific) according to manufacturer's instructions. Sequences were visualized and compared to the human reference sequence (GRCh37) using Sequence Pilot version 4.2.1 (JSI Medical Systems, Boston, MA).

Graphs with BMI trajectories were designed using Graph Pad Prism 7 (Graph Pad Software Inc., San Diego, CA, USA). Percentiles for BMI were drawn using the German growth reference data [11, 12]. Informed consent was obtained from the patients and also from the parents/guardians of the pediatric patients. The study was approved by the ethics committee of the University of Ulm (247/18) and complies with the declaration of Helsinki.

## Results

### Medical and family history

**Fig. 2:**
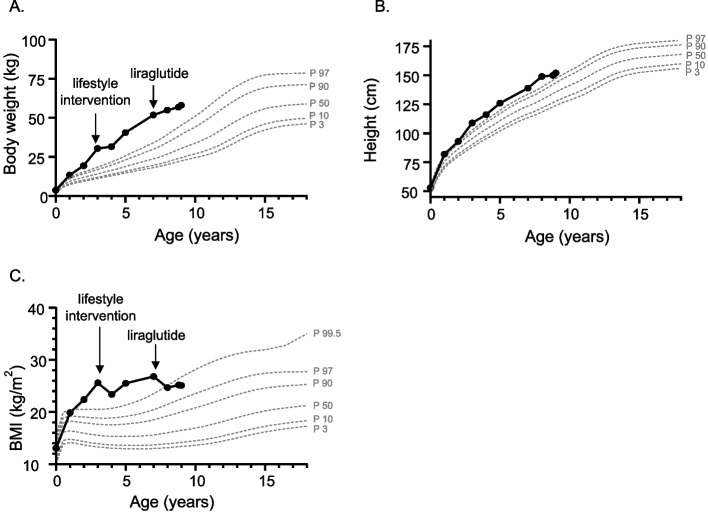
Trajectories over time of body weight (**A**), height (**B**) and body mass index (BMI) (**C**) of the index patient with monogenic obesity due to a monoallelic *MC4R* variant with corresponding interventions (lifestyle, liraglutide). Displayed in grey color are the percentiles for each somatometric measurement according to German growth reference data for girls [11, 12]

The index patient was referred to our clinic at the age of 7 years for further assessment due to severe early-onset obesity, marked hyperphagia and tall stature (Fig. 2). She was the second child of non-consanguineous parents that was born full-term following an unremarkable perinatal and postnatal course. Birth weight and length were within normal range. The patient exhibited normal psychomotor development, except for a mild speech delay. At presentation, she attended primary school, where she demonstrated strong academic engagement and performance. However, psychosocial stressors — particularly school-related teasing and bullying due to increased body weight — have contributed to progressive social withdrawal.

With respect to her obesity history, the patient demonstrated an intense drive for food intake beginning in the first months of life, leading to the onset of obesity by age of 1 year and progression to severe obesity by age of 2 years (Fig. 2). Because of the rapid weight gain and her hyperphagic behavior, an evaluation by a pediatric endocrinologist was performed at the age of 3 years in order to rule out a potential brain tumor. The diagnostic workup revealed no underlying etiology or associated comorbidities, and lifestyle modifications were recommended to manage weight gain. Adherence to dietary modifications and increased physical activity was effective in controlling weight gain, particularly during the initial phase. These lifestyle changes were maintained in the following years. Although weight gain continued, it occurred at a slower rate. As the patient grew taller, her BMI remained relatively stable (Fig. 2).

The patient’s family history revealed that multiple maternal relatives, including her mother, were affected by severe obesity evident from childhood (Fig. 1, Table 1). Notably, patient-reported histories indicate that the age of obesity onset decreased progressively across successive generations. Despite extensive clinical evaluations, a definitive etiological diagnosis had not been established. In detail, the mother of the index patient experienced a prolonged diagnostic and therapeutic odyssey beginning at the age of 6 years, which included among others participation in structured weight-loss programs at specialized obesity rehabilitation clinics and repeated attempts at calorie-restricted diets. Although these interventions resulted in temporary weight loss, weight regain occurred within months following each attempt. Controlling hunger and body weight became a central focus for both her and her daughter. Notably, while the index patient’s mother recalled experiencing marked hyperphagic behaviors during childhood, she reported no clinically significant hyperphagia in adulthood, as evidenced by stable eating patterns and absence of binge-eating episodes. The maternal grandmother of the index patient also presented with severe, early-onset obesity (Fig. 1). Like her daughter, multiple lifestyle interventions, initiated at the age of 12 years, were undertaken to manage weight gain but proved ineffective. In later adulthood, she developed several obesity-related comorbidities, including insulin resistance, dyslipidemia, and arterial hypertension (Table 1), in addition to fibromyalgia and depression. Finally, the index patient’s great-grandmother also experienced severe obesity beginning in late childhood. Interestingly, through sustained lifestyle modifications in adulthood, she achieved significant weight loss and currently maintains a normal BMI (Fig. 1).

**Table 1:** Clinical and laboratory characteristics of monoallelic carriers of the reported *MC4R* variant at presentation

	Index patient	Mother	Maternal grandmother	Maternal great-grandmother
Age (years)	7	39	64	87
BMI (SDS)	26.2 kg/m^2^ (+ 3.08)	54.7 kg/m^2^ (+ 2.89)	47.2 kg/m^2^ (+ 2.73)	22.2 kg/m^2^ (−1.30)
Childhood onset obesity	Yes	Yes	Yes	Yes
Hyperphagia	Yes	During childhood	No	No
Arterial hypertension	No	No	Yes	Yes
Type 2 diabetes mellitus	No	No	Impaired fasting glucose	No
Hyperinsulinaemia	No	No	Yes	N.A
MASLD	No	No	No	N.A
Dyslipidaemia	Yes	Yes	Yes	N.A

SDS for BMI was calculated according to German reference data [11, 12]

*BM* Body mass index, *SDS* Standard deviation score, *MASLD* Metabolic dysfunction-associated steatotic liver disease

### Clinical and laboratory findings

Physical examination of the index patient showed no signs of acanthosis nigricans and no evidence of pubertal onset. Laboratory investigations revealed elevated total cholesterol and LDL-cholesterol levels, while liver function and glucose metabolism, assessed via HbA1c and oral glucose tolerance test, were normal (Table 1). Given the clinical suspicion of monogenic obesity, genetic panel testing of six genes related to monogenic obesity was conducted and identified a rare monoallelic (heterozygous) variant in the *MC4R* gene (c.913C > T; p.(Arg305Trp), ENST00000299766). This variant has been described in clinical-genetic databases such as HGMD Professional, ClinVar, and LOVD, as well as in the literature, in monoallelic state in patients with obesity [17–19]. Experimental studies have shown that this missense variant impairs the function of MC4R [18, 20]. The in silico prediction programs included in the analysis to assess protein function impairment evaluated this variant controversially: while MetaRnn and AlphaMissense classify it as (likely) disease-causing, MetaLr and MetaSvm classify it as benign. In the population database gnomADv2.1.1, the variant is listed with a very low frequency of 0.002829%. Based on the ACMG guidelines [21], the variant is classified as a likely pathogenic variant of class 4.

### Follow-up

At the age of 8 years, treatment with glucagon-like peptide-1 (GLP-1) receptor agonist, liraglutide was initiated in the index patient, with gradual dose escalation to a maintenance dose of 3 mg/day. The treatment was well tolerated, and the patient demonstrated good adherence. During the first two years of therapy, notable improvements in hyperphagia and satiety were observed, along with a reduction in LDL-cholesterol levels. Weight gain was effectively managed, and with continued linear growth, a significant reduction in BMI was achieved (maximum BMI-SDS change −0.8 after two years of treatment, Fig. 2). The patient remains physically active, regularly engaging in sports activities.

The genetic diagnosis in the index patient prompted further genetic evaluation of additional family members affected by obesity, and confirmed the presence of the same *MC4R* variant in the mother, grandmother and great-grandmother of the index patient, but its absence in the brother, the father and the maternal grandfather (Fig. 1). Treatment with a GLP-1 receptor agonist was recommended to the mother and the grandmother of the index patient.

## Discussion

Monogenic obesity due to monoallelic *MC4R* variants can present with severe early-onset obesity yet often remains undiagnosed for generations. In the presented case, genetic testing in the youngest affected family member led to a diagnosis in three older relatives. This highlights the importance of genetic testing in children with severe obesity and hyperphagia, as it can guide targeted treatment and help establish a diagnosis for other family members.

MC4R is a G-protein coupled receptor with seven transmembrane domains that plays a central role in the leptin–melanocortin pathway, primarily regulating satiety, feeding behavior, and energy homeostasis [22, 23]. Expressed predominantly in the hypothalamus, MC4R is a key mediator of body weight regulation, and loss-of-function variants in this gene represent the most frequent cause of monogenic obesity [22, 24]. While biallelic MC4R variants are exceedingly rare, pathogenic monoallelic MC4R variants are present in up to 5.8% of individuals with severe, early-onset obesity [22, 25]. Most recently, a population-based study have found their prevalence at around 0.3% in a UK birth cohort [24], indicating that MC4R deficiency should no longer be classified as a “rare disease”.

The clinical manifestation of monoallelic MC4R deficiency shows considerable variability in obesity severity. Although biallelic carriers are rare and typically present with more severe phenotypes, certain individuals with monoallelic variants may remain unaffected by obesity [22, 24, 26, 27]. In addition to severe early-onset obesity and hyperphagia — the hallmark features — pathogenic *MC4R* variants have also been linked to accelerated linear growth during childhood (but normal final height), hyperinsulinemia, lower total and LDL-cholesterol levels, increased lean body mass and increased bone mineral density [10, 22, 28, 29]. Interestingly, both hyperinsulinemia and hyperphagia associated with MC4R deficiency appear to be age-dependent, tending to diminish over time, with the underlying mechanisms remaining unclear [22]. Earlier studies suggested that individuals with pathogenic *MC4R* variants may have lower blood pressure than noncarriers, but more recent findings from the same research group revealed no sustained reduction in blood pressure across the lifespan [24, 30]. In fact, a subsequent large study confirms that blood pressure in MC4R-deficient patients is largely comparable to BMI-matched controls, with the only exception being lower diastolic blood pressure observed in adolescents [10, 29]. Additionally, recent findings have identified macrocephaly as a common feature among individuals with *MC4R* deficiency [31], further expanding the phenotypic spectrum associated with these variants.

In the studied family, a clear pattern emerged across generations: severe obesity appeared at progressively younger ages, suggesting a progressive worsening of the phenotype. This observation aligns with previous research showing that the expressivity of obesity in individuals with loss-of-function *MC4R* variants tends to increase over generations, affecting grandparents, parents, and children with growing severity [19]. Unfortunately, in the present study historical BMI data from childhood or adulthood were not available for the affected family members other than the index patient, limiting the ability to compare growth trajectories and further investigate this trend. The mechanisms behind the observed generational trend remain unclear but likely involve both genetic and non-genetic factors, including the functional impact of *MC4R* variants, gene–gene interactions, and environmental influences [19, 26, 32]. Studies suggest that today’s obesogenic environment may exacerbate the effects of *MC4R* variants over time [19]. Other genetic influences may also shape outcomes. For instance, Chami et al. (25) showed that individuals with pathogenic *MC4R* variants and low polygenic risk may have milder phenotypes, highlighting overlap between monogenic and polygenic obesity [33]. Interestingly, in the present case one older affected family member achieved significant weight loss through lifestyle changes, despite carrying the same variant. This highlights the influence of modifiable factors and gene-environment interactions, reinforcing the need for further studies to clarify *MC4R* variant effects and guide personalized care [19, 26, 33].

Early identification and referral for genetic evaluation in children with suspected monogenic obesity are essential, as a confirmed diagnosis can reduce stigma, guide targeted treatment, and improve clinical outcomes [34]. A recent multicenter European study proposed characteristic BMI trajectories for children with genetic obesity due to biallelic variants and BMI cut-off values as a useful tool for considering genetic testing as well as for the follow-up of patients [10]. In adults, monogenic obesity is harder to recognize, but features such as early-onset obesity, hyperphagia, a family history of severe obesity, and related comorbidities (e.g., type 2 diabetes, elevated liver enzymes) should raise clinical suspicion [9]. Though universal screening is lacking, genetic panel testing is increasingly available in specialized centers nowadays.

Currently, there is no causal therapy for children with severe obesity due to MC4R deficiency. Early, targeted efforts focusing on diet and physical activity may help reduce long-term obesity and complication risk in *MC4R* variant carriers, but evidence is limited [24]. Multidisciplinary support, including psychological counseling, is key to effective care [34]. Some studies have shown that individuals with MC4R deficiency respond poorly to standard multidisciplinary lifestyle programs in terms of weight loss [35], while others report that weight loss is possible [36, 37], though maintaining it proves considerably more difficult for these patients [37].

New pharmacological options, however, show promise. GLP-1 receptor agonists like liraglutide and semaglutide have demonstrated potential for weight loss in this population, though existing studies are limited by small sample sizes [38, 39]. Recent investigations into dual-receptor agonists show further potential. A study of 31 adults with obesity due to MC4R deficiency indicated that tirzepatide, a dual GLP-1 and glucose-dependent insulinotropic polypeptide (GIP) receptor agonist, may be a particularly effective therapeutic strategy [40]. Setmelanotide, an MC4R agonist, has also shown efficacy in a subset of patients with *MC4R* variants, with ongoing research to further assess its benefits [41]. Given this variability in treatment response, individualized therapeutic approaches should be considered for patients with MC4R-related obesity. In the present study, liraglutide treatment was initiated off-label at the age of 8, following a confirmed genetic diagnosis and previous unsuccessful efforts to manage weight gain. The treatment was well tolerated and led to improved hunger control and stabilization of BMI over a 2-year period. Larger studies with extended follow-up are needed to evaluate the long-term efficacy and safety of GLP-1 analogues in children with monogenic obesity.

## Conclusion

This case demonstrates the critical role of early genetic testing in children with severe, early-onset obesity, which led to the identification of the same *MC4R* variant in four family members across four generations. While responses to treatment in monogenic obesity are variable, this case suggests GLP-1 analogues may benefit certain patients. Notably, we observed worsening obesity severity across successive generations, highlighting the compounding impact of today's obesogenic environment. Ultimately, a personalized, multidisciplinary approach remains essential to counteract these environmental pressures and improve outcomes for individuals with monogenic obesity.

## Data Availability

The dataset(s) supporting the conclusions of this article is(are) included within the article and its additional file(s). Further enquiries can be directed to the corresponding author.

## Abbreviations

LEP: Leptin
LEPR: Leptin receptor
MC4R: Melanocortin-4 receptor
POMC: Proopiomelanocortin
*PCSK1*: Proprotein convertase subtilisin/kexin type 1 gene
*SIM1*: Single-minded homolog 1 gene
*SH2B1*: SH2B adaptor protein 1 gene
*MRAP2*: Melanocortin receptor accessory protein 2 gene
BMI: Body mass index
SDS: Standard deviation score
GLP-1: Glucagon-like peptide-1

## References

[1] Styne DM, Arslanian SA, Connor EL, Farooqi IS, Murad MH, Silverstein JH, Yanovski JA (2017) Pediatric obesity-assessment, treatment, and prevention: an Endocrine Society clinical practice guideline. J Clin Endocrinol Metab 102(3):709–757. 10.1210/jc.2016-2573 28359099 10.1210/jc.2016-2573 PMC6283429

[2] Loos RJF, Yeo GSH (2022) The genetics of obesity: from discovery to biology. Nat Rev Genet 23(2):120–133. 10.1038/s41576-021-00414-z 34556834 10.1038/s41576-021-00414-z PMC8459824

[3] Ackerman GE, Smith ME, Mendelson CR, MacDonald PC, Simpson ER (1981) Aromatization of androstenedione by human adipose tissue stromal cells in monolayer culture. J Clin Endocrinol Metab 53(2):412–417. 10.1210/jcem-53-2-412 7251819 10.1210/jcem-53-2-412

[4] Hampl SE, Hassink SG, Skinner AC, Armstrong SC, Barlow SE, Bolling CF, Avila Edwards KC, Eneli I, Hamre R, Joseph MM, Lunsford D, Mendonca E, Michalsky MP, Mirza N, Ochoa ER, Sharifi M, Staiano AE, Weedn AE, Flinn SK, Okechukwu K (2023) Clinical practice guideline for the evaluation and treatment of children and adolescents with obesity. Pediatrics. 10.1542/peds.2022-06064037615077

[5] Argente J, Verge CF, Okorie U, Fennoy I, Kelsey MM, Cokkinias C, Scimia C, Lee HM, Farooqi IS (2025) Setmelanotide in patients aged 2–5 years with rare MC4R pathway-associated obesity (VENTURE): a 1 year, open-label, multicenter, phase 3 trial. Lancet Diabetes Endocrinol 13(1):29–37. 10.1016/s2213-8587(24)00273-0 39549719 10.1016/S2213-8587(24)00273-0

[6] Clément K, van den Akker E, Argente J, Bahm A, Chung WK, Connors H, De Waele K, Farooqi IS, Gonneau-Lejeune J, Gordon G, Kohlsdorf K, Poitou C, Puder L, Swain J, Stewart M, Yuan G, Wabitsch M, Kühnen P (2020) Efficacy and safety of setmelanotide, an MC4R agonist, in individuals with severe obesity due to LEPR or POMC deficiency: single-arm, open-label, multicentre, phase 3 trials. Lancet Diabetes Endocrinol 8(12):960–970. 10.1016/s2213-8587(20)30364-8 33137293 10.1016/S2213-8587(20)30364-8

[7] Farooqi IS, Jebb SA, Langmack G, Lawrence E, Cheetham CH, Prentice AM, Hughes IA, McCamish MA, O’Rahilly S (1999) Effects of recombinant leptin therapy in a child with congenital leptin deficiency. N Engl J Med 341(12):879–884. 10.1056/nejm199909163411204 10486419 10.1056/NEJM199909163411204

[8] Zorn S, von Schnurbein J, Kohlsdorf K, Denzer C, Wabitsch M (2020) Diagnostic and therapeutic odyssey of two patients with compound heterozygous leptin receptor deficiency. Mol Cell Pediatr 7(1):15. 10.1186/s40348-020-00107-3 33140236 10.1186/s40348-020-00107-3 PMC7606406

[9] Welling MS, Mohseni M, Meeusen REH, de Groot CJ, Boon MR, Kleinendorst L, Visser JA, van Haelst MM, van den Akker ELT, van Rossum EFC (2024) Clinical phenotypes of adults with monogenic and syndromic genetic obesity. Obesity 32(7):1257–1267. 10.1002/oby.24047 38807300 10.1002/oby.24047

[10] Zorn S, de Groot CJ, Brandt-Heunemann S, von Schnurbein J, Abawi O, Bounds R, Ruck L, Guijo B, Martos-Moreno G, Nicaise C, Courbage S, Klehr-Martinelli M, Siebert R, Dubern B, Poitou C, Clément K, Argente J, Kühnen P, Farooqi IS, van den Akker E (2025) Early childhood height, weight, and BMI development in children with monogenic obesity: a European multicentre, retrospective, observational study. Lancet Child Adolesc Health 9(5):297–305. 10.1016/s2352-4642(25)00065-3 40246357 10.1016/S2352-4642(25)00065-3

[11] Kromeyer-Hauschild K, Moss A, Wabitsch M (2015) Referenzwerte für den Body-Mass-Index für Kinder, Jugendliche und Erwachsene in Deutschland [Body mass index reference values for German children, adolescents and adults]. Anpassung der AGA-BMI-Referenz im Altersbereich von 15 bis 18 Jahren. 09 03:123–127. 10.1055/s-0037-1618928

[12] Kromeyer-Hauschild K, Wabitsch M, Kunze D, Geller F, Geiß HC, Hesse V, von Hippel A, Jaeger U, Johnsen D, Korte W, Menner K, Müller G, Müller JM, Niemann-Pilatus A, Remer T, Schaefer F, Wittchen HU, Zabransky S, Zellner K, Hebebrand J (2001) Perzentile für den Body-mass-Index für das Kindes- und Jugendalter unter Heranziehung verschiedener deutscher Stichproben. Monatsschr Kinderheilkd 149(8):807–818. 10.1007/s001120170107

[13] Dykens EM, Maxwell MA, Pantino E, Kossler R, Roof E (2007) Assessment of hyperphagia in Prader-Willi syndrome. Obesity (Silver Spring) 15(7):1816–1826. 10.1038/oby.2007.216 17636101 10.1038/oby.2007.216

[14] de Ferranti SD, Steinberger J, Ameduri R, Baker A, Gooding H, Kelly AS, Mietus-Snyder M, Mitsnefes MM, Peterson AL, St-Pierre J, Urbina EM, Zachariah JP, Zaidi AN (2019) Cardiovascular risk reduction in high-risk pediatric patients: a scientific statement from the American Heart Association. Circulation 139(13):e603–e634. 10.1161/cir.0000000000000618 30798614 10.1161/CIR.0000000000000618

[15] (2011) Expert panel on integrated guidelines for cardiovascular health and risk reduction in children and adolescents: summary report. Pediatrics 128 Suppl 5(Suppl 5):S213–256. 10.1542/peds.2009-2107C 10.1542/peds.2009-2107C PMC4536582 22084329

[16] Grundy SM, Stone NJ, Bailey AL, Beam C, Birtcher KK, Blumenthal RS, Braun LT, de Ferranti S, Faiella-Tommasino J, Forman DE, Goldberg R, Heidenreich PA, Hlatky MA, Jones DW, Lloyd-Jones D, Lopez-Pajares N, Ndumele CE, Orringer CE, Peralta CA, Saseen JJ, Smith SC Jr, Sperling L, Virani SS, Yeboah J (2019) 2018 AHA/ACC/AACVPR/AAPA/ABC/ACPM/ADA/AGS/APhA/ASPC/NLA/PCNA guideline on the management of blood cholesterol: a report of the American College of Cardiology/American Heart Association Task Force on Clinical Practice Guidelines. Circulation 139(25):e1082–e1143. 10.1161/cir.0000000000000625 30586774 10.1161/CIR.0000000000000625 PMC7403606

[17] Kleinendorst L, Abawi O, van der Voorn B, Jongejan M, Brandsma AE, Visser JA, van Rossum EFC, van der Zwaag B, Alders M, Boon EMJ, van Haelst MM, van den Akker ELT (2020) Identifying underlying medical causes of pediatric obesity: results of a systematic diagnostic approach in a pediatric obesity center. PLoS One 15(5):e0232990. 10.1371/journal.pone.0232990 32384097 10.1371/journal.pone.0232990 PMC7209105

[18] Lubrano-Berthelier C, Dubern B, Lacorte JM, Picard F, Shapiro A, Zhang S, Bertrais S, Hercberg S, Basdevant A, Clement K, Vaisse C (2006) Melanocortin 4 receptor mutations in a large cohort of severely obese adults: prevalence, functional classification, genotype-phenotype relationship, and lack of association with binge eating. J Clin Endocrinol Metab 91(5):1811–1818. 10.1210/jc.2005-1411 16507637 10.1210/jc.2005-1411

[19] Stutzmann F, Tan K, Vatin V, Dina C, Jouret B, Tichet J, Balkau B, Potoczna N, Horber F, O’Rahilly S, Farooqi IS, Froguel P, Meyre D (2008) Prevalence of melanocortin-4 receptor deficiency in Europeans and their age-dependent penetrance in multigenerational pedigrees. Diabetes 57(9):2511–2518. 10.2337/db08-0153 18559663 10.2337/db08-0153 PMC2518504

[20] Roubert P, Dubern B, Plas P, Lubrano-Berthelier C, Alihi R, Auger F, Deoliveira DB, Dong JZ, Basdevant A, Thurieau C, Clément K (2010) Novel pharmacological MC4R agonists can efficiently activate mutated MC4R from obese patient with impaired endogenous agonist response. J Endocrinol 207(2):177–183. 10.1677/joe-09-0336 20696697 10.1677/JOE-09-0336

[21] Richards S, Aziz N, Bale S, Bick D, Das S, Gastier-Foster J, Grody WW, Hegde M, Lyon E, Spector E, Voelkerding K, Rehm HL (2015) Standards and guidelines for the interpretation of sequence variants: a joint consensus recommendation of the American College of Medical Genetics and Genomics and the Association for Molecular Pathology. Genet Med 17(5):405–424. 10.1038/gim.2015.30 25741868 10.1038/gim.2015.30 PMC4544753

[22] Farooqi IS, Keogh JM, Yeo GS, Lank EJ, Cheetham T, O’Rahilly S (2003) Clinical spectrum of obesity and mutations in the melanocortin 4 receptor gene. N Engl J Med 348(12):1085–1095. 10.1056/NEJMoa022050 12646665 10.1056/NEJMoa022050

[23] Namjou B, Stanaway IB, Lingren T, Mentch FD, Benoit B, Dikilitas O, Niu X, Shang N, Shoemaker AH, Carey DJ, Mirshahi T, Singh R, Nestor JG, Hakonarson H, Denny JC, Crosslin DR, Jarvik GP, Kullo IJ, Williams MS (2021) Evaluation of the MC4R gene across eMERGE network identifies many unreported obesity-associated variants. Int J Obes 45(1):155–169. 10.1038/s41366-020-00675-4 10.1038/s41366-020-00675-4 PMC7752751 32952152

[24] Wade KH, Lam BYH, Melvin A, Pan W, Corbin LJ, Hughes DA, Rainbow K, Chen JH, Duckett K, Liu X, Mokrosiński J, Mörseburg A, Neaves S, Williamson A, Zhang C, Farooqi IS, Yeo GSH, Timpson NJ, O’Rahilly S (2021) Loss-of-function mutations in the melanocortin 4 receptor in a UK birth cohort. Nat Med 27(6):1088–1096. 10.1038/s41591-021-01349-y 34045736 10.1038/s41591-021-01349-y PMC7611835

[25] Vollbach H, Brandt S, Lahr G, Denzer C, von Schnurbein J, Debatin KM, Wabitsch M (2017) Prevalence and phenotypic characterization of MC4R variants in a large pediatric cohort. Int J Obes (Lond) 41(1):13–22. 10.1038/ijo.2016.161 27654141 10.1038/ijo.2016.161

[26] Tao YX, Segaloff DL (2005) Functional analyses of melanocortin-4 receptor mutations identified from patients with binge eating disorder and nonobese or obese subjects. J Clin Endocrinol Metab 90(10):5632–5638. 10.1210/jc.2005-0519 16030156 10.1210/jc.2005-0519

[27] Vaisse C, Clement K, Durand E, Hercberg S, Guy-Grand B, Froguel P (2000) Melanocortin-4 receptor mutations are a frequent and heterogeneous cause of morbid obesity. J Clin Invest 106(2):253–262. 10.1172/jci9238 10903341 10.1172/JCI9238 PMC314306

[28] Farooqi IS (2022) Monogenic obesity syndromes provide insights into the hypothalamic regulation of appetite and associated behaviors. Biol Psychiatry 91(10):856–859. 10.1016/j.biopsych.2022.01.018 35369984 10.1016/j.biopsych.2022.01.018

[29] Zorn S, Bounds R, Williamson A, Lawler K, Hanssen R, Keogh J, Henning E, Smith M, Fielding BA, Umpleby AM, Yasmeen S, Marti-Solano M, Langenberg C, Wabitsch M, Collet TH, Farooqi IS (2025) Obesity due to MC4R deficiency is associated with reduced cholesterol, triglycerides and cardiovascular disease risk. Nat Med. 10.1038/s41591-025-03976-1 10.1038/s41591-025-03976-1 PMC12705457 41102563

[30] Greenfield JR, Miller JW, Keogh JM, Henning E, Satterwhite JH, Cameron GS, Astruc B, Mayer JP, Brage S, See TC, Lomas DJ, O’Rahilly S, Farooqi IS (2009) Modulation of blood pressure by central melanocortinergic pathways. N Engl J Med 360(1):44–52. 10.1056/NEJMoa0803085 19092146 10.1056/NEJMoa0803085

[31] van der Walle E, de Groot CJ, Kleinendorst L, de Klerk H, Welling MS, Abawi O, Meeusen REH, Boon MR, van Rossum EFC, van Haelst MM, van den Akker ELT (2025) Unraveling the relationship between head circumference and MC4R deficiency from infancy to adulthood: a case-control study. Obesity (Silver Spring) 33(5):986–995. 10.1002/oby.24263 40231439 10.1002/oby.24263 PMC12015652

[32] Dempfle A, Hinney A, Heinzel-Gutenbrunner M, Raab M, Geller F, Gudermann T, Schäfer H, Hebebrand J (2004) Large quantitative effect of melanocortin-4 receptor gene mutations on body mass index. J Med Genet 41(10):795–800. 10.1136/jmg.2004.018614 15466016 10.1136/jmg.2004.018614 PMC1735595

[33] Chami N, Preuss M, Walker RW, Moscati A, Loos RJF (2020) The role of polygenic susceptibility to obesity among carriers of pathogenic mutations in MC4R in the UK Biobank population. PLoS Med 17(7):e1003196. 10.1371/journal.pmed.1003196 32692746 10.1371/journal.pmed.1003196 PMC7373259

[34] Schirmer M, Zorn S, von Schnurbein J, Wabitsch M (2025) Innovative care for children and adolescents with severe and/or genetic obesity. Horm Res Paediatr 1–10. 10.1159/000544177 10.1159/000544177 40159376

[35] Trier C, Hollensted M, Schnurr TM, Lund MAV, Nielsen TRH, Rui G, Andersson EA, Svendstrup M, Bille DS, Gjesing AP, Fonvig CE, Frithioff-Bøjsøe C, Balslev-Harder M, Quan S, Gamborg M, Pedersen O, Ängquist L, Holm JC, Hansen T (2021) Obesity treatment effect in Danish children and adolescents carrying Melanocortin-4 receptor mutations. Int J Obes (Lond) 45(1):66–76. 10.1038/s41366-020-00673-6 32921795 10.1038/s41366-020-00673-6 PMC7752754

[36] Hainerová I, Larsen LH, Holst B, Finková M, Hainer V, Lebl J, Hansen T, Pedersen O (2007) Melanocortin 4 receptor mutations in obese Czech children: studies of prevalence, phenotype development, weight reduction response, and functional analysis. J Clin Endocrinol Metab 92(9):3689–3696. 10.1210/jc.2007-0352 17579204 10.1210/jc.2007-0352

[37] Reinehr T, Hebebrand J, Friedel S, Toschke AM, Brumm H, Biebermann H, Hinney A (2009) Lifestyle intervention in obese children with variations in the melanocortin 4 receptor gene. Obesity (Silver Spring) 17(2):382–389. 10.1038/oby.2008.422 18997677 10.1038/oby.2008.422

[38] Gokul PR, Apperley L, Parkinson J, Clark K, Lund K, Owens M, Senniappan S (2025) Semaglutide, a long-acting GLP-1 analogue, for the management of early-onset obesity due to MC4R defect: a case report. Horm Res Paediatr 98(2):148–155. 10.1159/000537921 38402868 10.1159/000537921

[39] Iepsen EW, Zhang J, Thomsen HS, Hansen EL, Hollensted M, Madsbad S, Hansen T, Holst JJ, Holm JC, Torekov SS (2018) Patients with obesity caused by melanocortin-4 receptor mutations can be treated with a glucagon-like peptide-1 receptor agonist. Cell Metab 28(1):23-32.e23. 10.1016/j.cmet.2018.05.008 29861388 10.1016/j.cmet.2018.05.008

[40] Bhatnagar P, Ahmad NN, Li X, Coghlan M, Kaplan LM, Farooqi IS (2025) Tirzepatide leads to weight reduction in people with obesity due to MC4R deficiency. Nat Med. 10.1038/s41591-025-03913-2 40858971 10.1038/s41591-025-03913-2 PMC12532586

[41] Collet TH, Dubern B, Mokrosinski J, Connors H, Keogh JM, de Mens Oliveira E, Henning E, Poitou-Bernert C, Oppert JM, Tounian P, Marchelli F, Alili R, Le Beyec J, Pépin D, Lacorte JM, Gottesdiener A, Bounds R, Sharma S, Folster C, Van der Ploeg LHT (2017) Evaluation of a melanocortin-4 receptor (MC4R) agonist (Setmelanotide) in MC4R deficiency. Mol Metab 6(10):1321–1329. 10.1016/j.molmet.2017.06.015 29031731 10.1016/j.molmet.2017.06.015 PMC5641599

